# HIV/HCV co-infection and associated risk factors among injecting drug users in Dar es Salaam, Tanzania: potential for HCV elimination

**DOI:** 10.1186/s12954-019-0346-y

**Published:** 2019-12-11

**Authors:** Germana Henry Leyna, Neema Makyao, Alexander Mwijage, Angela Ramadhan, Samuel Likindikoki, Mucho Mizinduko, Melkizedeck Thomas Leshabari, Kåre Moen, Elia John Mmbaga

**Affiliations:** 1 Department of Epidemiology and Biostatistics, Muhimbili University of Health and Allied Sciences, P.O. Box 65015, Dar es Salaam, Tanzania; 2 Ministry of Health, Community Development, Gender, Elderly and Children, Dodoma, Tanzania; 3 University of Oslo, Oslo, Norway

**Keywords:** HCV, HIV, Injecting drugs, Risk factors, Elimination, Tanzania

## Abstract

**Background:**

Chronic HCV infection causes substantial morbidity and mortality and, in co-infection with HIV, may result in immunological and virological failure following antiretroviral treatment. Estimates of HCV infection, co-infection with HIV and associated risk practices among PWID are scarce in Africa. This study therefore aimed at estimating the prevalence of HCV and associated risk factors among PWID in the largest metropolitan city in Tanzania to inform WHO elimination recommendations.

**Methods:**

An integrated bio-behavioral survey using respondent-driven sampling was used to recruit PWID residing in Dar es Salaam, Tanzania. Following face-to-face interviews, blood samples were collected for HIV and HCV testing. Weighted modified Poisson regression modeling with robust standard errors was used in the analysis.

**Results:**

A total of 611 PWID with a median age of 34 years (IQR, 29–38) were recruited through 4 to 8 waves. The majority of participants (94.3%) were males, and the median age at first injection was 24 years (IQR, 19–30). Only 6.55% (40/611) of participants reported to have been enrolled in opioid treatment programs. The weighted HCV antibody prevalence was 16.2% (95%CI, 13.0–20.1). The corresponding prevalence of HIV infection was 8.7% (95%CI, 6.4–11.8). Of the 51 PWID who were infected with HIV, 22 (43.1%) were HCV seropositive. Lack of access to clean needles (adjusted prevalence ratio (APR), 1.76; 95%CI, 1.44; 12.74), sharing a needle the past month (APR, 1.72; 95%CI, 1.02; 3.00), not cleaning the needle the last time shared (APR, 2.29; 95%CI, 1.00; 6.37), and having unprotected not using a transactional sex (APR, 1.87; 95%CI, 1.00; 3.61) were associated with increased risk of HCV infection. On the other hand, not being on opioid substitution therapy was associated with 60% lower likelihood of infection.

**Conclusions:**

The HCV antibody prevalence among PWID is lower than global estimates indicating potential for elimination. Improving access to safe injecting paraphernalia, promoting safer injecting practices is the focus of prevention programing. Screening for HIV/HCV co-infection should be intensified in HIV care, opioid substitution programs, and other point of care for PWID. Use of direct-acting antiretroviral treatment would accelerate the achievement of hepatitis infection elimination goal by 2030.

## Background

Chronic hepatitis C virus (HCV) infection is a major health problem affecting approximately 185 million people globally with people who inject drugs (PWID) bearing the brunt of the epidemic [1–3]. HCV prevalence among PWID varies widely but it is estimated that 52.3% of the 15.6 million PWID globally are HCV seropositive with an estimated incidence rate of 5 to 45% [1]. 

While infection with HCV has been attributed to development of chronic infection in 80% of cases, up to 11% of those with chronic infection develop liver cirrhosis within 20 years with potential for liver failure and hepatocellular carcinoma [4, 5]. In sub-Saharan Africa where the rate of HIV infection is high and the fact that HIV and HCV share some transmission risk factors such as injecting behaviors, the potential for HIV/HCV co-infection is extremely high [6–8]. HIV/HCV co-infection results into accelerated hepatic fibrosis, higher rates of liver decompensation and death compared to HCV mono-infection [7, 9, 10].

A facility-based study involving 630 PWID attending opioid substation therapy (OST) in Dar es Salaam, Tanzania, revealed that more than half (57%) of PWID were seropositive for HCV [11]. A recent analysis of data from 1350 PWID attending OST indicated that, of the HCV seropositive PWID, 44% were co-infected with HIV [12].

Direct-acting antiretroviral (DAA) treatments have changed HCV to be an easily curable infection [13–15]. Studies have indicated that treatment of all persons infected with HCV with DAA is cost-effective and increases potential for elimination [14, 16]. In 2018, the World Health Organization (WHO) released a guideline for the care and treatment of persons diagnosed with chronic hepatitis C Virus Infection to facilitate the global goals of eliminating HCV by 2030 [15].

Modeling studies have indicated that prevention impact to be achieved by initiating the WHO recommendation depends on the prevalence of HCV among PWID [13]. This study therefore aimed at estimating the prevalence of HCV infection, HIV/HCV co-infection, and associated risk factors among PWID in the largest metropolitan city of Dar es Salaam, Tanzania, to inform elimination programming.

## Methods

### Design and setting

This was a cross-sectional integrated bio-behavioral survey (IBBS) of PWID living in Dar es Salaam, Tanzania. The study was conducted between September and December 2017 recruiting PWID from five municipalities of Dar es Salaam, the largest metropolitan city in Tanzania, with an estimated population of 5 million people. A recent IBBS indicate that the city has substantial number of PWID with HIV prevalence as high as 15% compare to the general population estimate of 4.7% among adult aged 15–64 in the city [6, 17].

### Study population

Participants were eligible if they were aged 18 and above, had injected drugs during the past 3 months, and resident of any of the five municipalities of Dar es Salaam. In this study, residence was defined as having an address in the city and having lived in the city for the past 6 months preceding fieldwork.

### Power and sample size estimation

Power and sample size estimates were based on the estimates for HIV infection among PWID of 15.5% from a survey conducted in 2014 [6]. With a precision of 5% and design effect (DEFF) of 3, based on the median DEFF found for key variables in similar previous surveys in the country, the minimum sample size was estimated to be 601.

### Sampling and recruitment

Respondent-driven sampling (RDS), a method developed for the sampling from populations for which there is no available sampling frame, was used to recruit study participants. RDS is based on the principle that members of the target population refer other members of the same population to participate, so that the sample is established by successive generations of recruitment referrals. RDS builds on a mathematical model which provides a theoretical basis for estimation of population proportions and their variances through statistical adjustment [18, 19].

### Data collection tool

Previously used questionnaire for surveys of PWID were adapted and used in this survey [6]. The questionnaire was pre-tested among peer educators before implementation in the field to assure that language, cultural, and peer norms are considered. All questions were translated from English to Swahili and translated back to English to ensure face validity. The questionnaire was uploaded onto the Open Data Kit (ODK) platform and assessed for consistency. All information for each participant was linked to a coupon number given to each participant. Each participant had to have a coupon to participate. In the formative assessment phase, we assessed the acceptability of using electronic data collection methods for data collection and to alleviate any misconceptions on use of these device such as taking images or recordings.

### Data collection procedures

A total of 5 seeds were recruited from different client meeting points through contacts made by peer educators. Seed selection was done to ensure that PWID from different socioeconomic groups, sex, locality, and education levels are represented. Before recruitment, participants were screened for eligibility by a trained screener, a former PWID who stopped injecting after enrolment into OST. Upon giving informed consent, face-to-face interviews using a tablet were conducted by trained research assistants. Following the face-to-face interview, participants who also consented to biological testing received pre-test counseling for HIV, and hepatitis following the National guidelines for HIV testing and counseling in clinical settings. A qualified and trained phlebotomist collected 10 ml of venous blood from the left arm following standard blood collection procedures. All collected specimen were labeled with the participant’s coupon identification number, which served as the link between the specimen and the participant’s completed questionnaire. After completion of data collection activities, each recruiter received three recruitment coupons with which to recruit their peers into the survey. Enrolled seeds recruited the first wave of participants for the survey. Participants received compensation for transport to come to the interview venue amounting into approximately US$ 4 and they also received approximately US$ 2 for each of the three fellow PWID recruited. The rate for this reimbursement agreed upon by the key population for HIV advisory board which includes a PWID and was approved by the ethic review committee.

### Laboratory testing

HIV testing was done with SD Bioline HIV-1/2 3.0 rapid test (Standard Diagnostics, Inc., Korea). Non-reactive results were considered negative for HIV antibodies whereas reactive results were subsequently tested with Uni-GoldTM HIV-1/2 (Trinity Biotech Plc, Ireland). Discrepant results between the first and second rapid assays were resolved by Enzygnost HIV Integral 4 (Siemens, Germany). Presence of hepatitis B and C antibodies were tested using Murex HBsAg version 3 ELISA (Diasorin, UK) and Murex HCV ELISA (Diasorin, UK) respectively.

### Data analysis

Data analysis was carried out using STATA version 15 and RDSAT statistical packages. Use of RDS result into different selection probability for each participant with larger networks is more likely to be represented than smaller ones. Data were therefore weighted by calculating weight as an inverse of the participant’s network size and controlled clustering by multiplying the weight by the sample size and divided it by the sum of the weights. Categorical variables were summarized by calculating proportions and differences in proportion examined using *χ*2 test. Median and interquarter range (IQR) were used to summarize continuous variables. Because of higher prevalence of the outcome variable (HCV infection), we employed weighted modified Poisson regression with robust standard error to estimate crude prevalence ratio (PR) instead of conventional logistic regression which would have overestimated the odds ratio. A stepwise-backward elimination weighted modified multivariable Poisson regression models with robust standard errors were built to identify independent predictors of HCV infection. The best parsimonious model was based on the lowest Akaike Information criteria (AIC). All analyses were two-tailed and the significance level was set at 5%.

## Results

A total of 611 eligible PWID were recruited through 4 to 8 recruitment waves during the data collection period. Majority of those who participated were male (94.3%, 576/611) with an overall median age of 34 years (IQR, 29–38). Majority of the participants (73.0%, 446/611) reported to have completed primary education, half (52.9%, 323/611) never married, 71.5% (437/611) were born and raised in the city of Dar es Salaam, and half (48.6%, 297/611) lived with their family. Self-employment including petty trading was the main source of income (73.7%, 450/611) and the median monthly income was Tanzania Shillings (TZS) 150,000 (IQR, 80,000–300,000) (Table 1).

**Table 1:** Comparison of sociodemographic characteristics by HCV Infection among PWID in Dar es Salaam, Tanzania

Variable	Category	*n* (%)	HCV infection		*p* value
			Positive *n* (%)	Negative *n* (%)	
Age groups (years)	15–24	54 (8.84)	4 (7.41)	50 (92.59)	0.181
Sex	25–34	267 (83	42 (15.91)	222 (84.09)	
	35 and above	29 (47.46)	50 (17.42)	237 (82.58)	
	Male	576 (94.27)	91 (15.91)	481 (84.09)	0.908
	Female	35 (5.73)	5 (15.15)	29 (84.85)	
Occupation	None	61 (9.98)	14 (23.33)	46 (76.67)	0.082
	Petty/self-employed	450 (73.65)	72 (16.14)	374 (83.86)	
	Employed	100 (16.37)	10 (10.10)	89 (89.90)	
Education level	No formal	32 (5.23)	5 (16.13)	26 (83.87)	0.999
	Primary	446 (73.00)	70 (15.84)	372 (84.87)	
	Secondary and above	133 (21.77)	21 (15.91)	111 (84.09)	
Marital Status	Never married	323 (52.86)	47 (14.46)	274 (85.36)	0.117
	Married/cohabiting	115 (18.82)	14 (12.28)	100 (87.72)	
	Divorced/separated	173 (28.31)	35 (20.59)	135 (79.41)	
Time lived in Dar es Salaam	Born and raised	437 (71.52)	65 (15.05)	367 (84.95)	0.382
	Not born	174 (28.48)	31 (17.92)	142 (82.08)	
Income past month (TZS)	< 50,000	116 (18.99)	14 (12.07)	102 (87.93)	0.175
	50,000–120,000	145 (23.73)	25 (17.36)	119 (82.64)	
	121,000–200,000	95 (15.55)	10 (10.64)	84 (89.36)	
	> 200,000	255 (41.73)	47 (18.73)	204 (81.27)	
Currently living with	Alone	181 (29.62)	30 (16.67)	150 (83.33)	0.434
	Family	297 (48.61)	51 (17.41)	242 (82.59)	
	Partner	101 (16.53)	12 (12.00)	88 (88.00)	
	Friends	32 (5.24)	3 (9.38)	29 (90.63)	

### Risk injecting and sexual behaviors

Unsafe injecting practices and other risk behaviors for HCV infection in this population were common. The median age at first injection was 24 years (IQR, 19–30). Of the 611 participants, 486 (76.6%) reported to inject drugs several times per day and nearly a third (29.8%, 182/611) did not have access to clean needles every time they needed one. While 41.4% (253/611) reported to have ever shared a needle, 68.4% of those (173/253) shared a needle during the past month and 67.1% (116/173) of those shared past months did not wash a needle before injecting (Table 1). With regards to sexual behaviors, nearly three quarter (69.1%, 422/611) reported to have ever sold sex and about half (48.1%, 203/422) did so during the last month preceding the survey. A large majority (70.0% 142/203) of those who sold sex last month did not use a condom. Being exposed to physical violence (beaten) was reported by 37.1% (227/611) of the participants and 14.9% (91/611) reported sexual violence involvement. Only a third of the participants had comprehensive knowledge of HIV transmission and prevention (36.8%, 225/611) and while almost similar proportion perceived themselves to be at lower or no risk of infection (32.9%, 201/611). Of all the study participants, only 6.55% (40/611) reported to have been enrolled in Opioid Treatment Program (Tables 1 and 2).

**Table 2:** Comparison of injecting and other Risk characteristics by HCV Infection among PWID in Dar es Salaam, Tanzania

Variable	Category	*n* (%)	HCV infection		*p* value
			Positive *n* (%)	Negative *n* (%)	
Age at first injection	≤ 15	57 (9.33)	12 (21.05)	45 (78.95)	0.334
	16–18	79 (12.93)	15 (18.99)	64 (81.01)	
	> 18	475 (77.74)	69 (14.71)	400 (85.29)	
Duration of injection (years)	< 1	47 (7.69)	3 (6.38)	44 (93.62)	
	1–3	108 (17.68)	13 (12.26)	93 (87.74)	0.034
	4–6	117 (19.15)	13 (11.21)	103 (88.79)	
	7–9	79 (12.44)	16 (21.33)	59 (78.67)	
	10+	263 (43.04)	51 (19.54)	210 (80.46)	
Injection past month, how often	Once/day	19 (3.11)	1 (5.26)	18 (94.74)	
	> 1 per day	468 (76.60)	78 (16.85)	385 (83.15)	0.506
	Once/week	50 (8.18)	7 (14.29)	42 (85.71)	
	> 1 per week	74 (12.11)	10 (13.51)	64 (86.49)	
Access to clean needle when needed	Yes	429 (70.21)	62 (14.55)	355 (85.45)	0.042
	No	182 (29.79)	34 (18.99)	154 (81.01)	
Flash blood past month*	Yes	28 (4.58)	2 (7.41)	25 (92.59)	0.218
	No	583 (95.42)	94 (16.26)	484 (83.74)	
Ever shared needle	Yes	253 (41.41)	41 (16.47)	208 (83.53)	0.736
	No	358 (58.59)	55 (15.45)	301 (84.55)	
Shared needle past month	Yes	173 (68.38)	33 (18.33)	147 (81.67)	0.007
	No	80 (31.62)	8 (11.59)	61 (88.41)	
Cleaned needle last time shared	Yes	57 (32.95)	3 (6.00)	47 (94.00)	0.026
	No	116 (69.05)	38 (19.10)	161 (80.90)	
On opioid treatment Program	Yes	40 (6.55)	14 (35.90)	25 (64.10)	< 0.001
	No	571 (93.45)	82 (14.49)	484 (85.51)	
Ever sold sex	Yes	422 (69.07)	70 (16.71)	349 (83.29)	0.397
	No	189 (30.93)	26 (13.98)	160 (86.02)	
Sold sex past month	Yes	203 (48.10)	30 (14.85)	172 (85.15)	0.326
	No	219 (51.90)	40 (18.43)	177 (81.57)	
Condom use last paid sex	Yes	61 (30.05)	5 (8.20)	56 (91.80)	0.080
	No	142 (69.95)	25 (17.73)	116 (82.27)	
Ever beaten past year	Yes	227 (37.15)	34 (15.11)	191 (84.89)	0.695
	No	384 (62.85)	62 (16.32)	318 (83.68)	
Forced sex past year	Yes	91 (14.89)	10 (11.11)	80 (88.89)	0.181
	No	520 (85.11)	86 (16.70)	429 (83.30)	
Ever arrested past year	Yes	358 (58.59)	49 (19.52)	202 (80.48)	0.038
	No	253 (41.41)	47 (13.28)	307 (86.72)	
Comprehensive HIV knowledge	Yes	225 (36.82)	34 (15.18)	190 (84.82)	0.722
	No	386 (63.18)	62 (16.27)	319 (83.73)	
Risk perception	No or low	201 (32.90)	31 (15.50)	169 (84.50)	0.662
	Moderate	92 (15.06)	17 (19.10)	72 (80.90)	
	High	318 (52.05)	48 (15.19)	268 (84.81)	

### Prevalence of HCV infection and HIV/HCV co-infection

The weighted HCV antibody prevalence in the population was 16.2% (95%CI, 13.0–20.1). The corresponding prevalence of HIV infection was 8.7% (95%CI, 6.4–11.8). Of the 51 PWID who were infected with HIV, 22 (43.1%) tested positive for HCV infection. The rate of HIV/HCV co-infection was significantly higher among men as compared to female (50.0% versus 23.1%, *p* < 0.001) and those aged above 35 as compared to those aged 15–24 (52.6% versus 0.0%, *p* < 0.001).

While infection with HCV among PWID in Dar es Salaam did not vary by sociodemographic characteristics, the prevalence increased with years of injection (*p* = 0.034), lack of easy access to clean needles (18.99% versus 14.55%, *p* = 0.042), sharing needles the past month (18.33% versus 11.59, *p* = 0.007), not cleaning needle the last time it was shared (19.10% versus 6.00%, *p* = 0.026), and reporting to ever been arrested the past year (19.52% versus 13.28%, *p* = 0.038) (Table 2).

### Factors associated with HCV infection

Crude modified Poisson regression of factors associated with HCV infection revealed that PWID who have injected for 7 years or more were associated with a three-fold increased risk of HCV infection. Reported lack of access to clean needles when in need (PR, 1.83; 95%CI, 1.54; 11.27), shared needle last month (PR, 1.68; 95%CI, 1.00; 2.94), not cleaning a needle the last time it was shared (PR, 2.27; 95%CI, 1.01; 6.38) were significantly associated with increased risk of HCV infection. On the other hand, PWID who were not been enrolled on Opioid Treatment Program had a 60% lower risk of HCV infection as compared to those enrolled in the program (PR, 0.40; 95%CI, 0.25; 0.64) (Tables 3 and 4).

**Table 3:** Modified Poisson regression modeling of socio-demographic risk factors for HCV infection among PWID in Dar es Salaam, Tanzania

Variable	Category	PR (95%CI)	APR (95%CI)	*p* value
Age groups (years)	15–24	1	1	
	25–34	2.14 (0.80; 5.74)	2.11 (0.81; 5.76)	0.129
	35 and above	2.35 (0.88; 6.24	2.35 (0.89; 6.27)	0.088
Sex	Male	1	1	
	Female	0.95 (0.41; 2.18)	0.98 (0.43; 2.25)	0.950
Education level	No formal	1	1	
	Primary	0.98 (0.42; 2.25)	0.97 (0.43; 2.22)	0.950
	Secondary and above	0.99 (0.40; 2.41)	0.97 (0.40; 2.36)	0.954
Marital status	Never married	1	1	
	Married/cohabiting	0.84 (0.48; 1.46)	0.81 (0.46; 1.41)	0.456
	Divorced/separated	1.40 (0.94; 2.09)	1.31 (0.87; 1.98)	0.201
Time lived in Dar es Salaam	Born and raised	1	1	
	Not born	1.19 (0.80; 1.75)	1.20 (0.82; 1.78)	0.342
Income past month (TZS)	< 50,000	1	1	
	50,000–120,000	1.44 (0.78; 2.64)	1.42 (0.77; 2.60)	0.253
	121,000–200,000	0.88 (0.41; 1.89)	0.83 (0.38; 1.82)	0.636
	> 200,000	1.55 (0.89; 2.70)	1.50 (0.86; 2.62)	0.152
Currently living with	Alone	1	1	
	Family	1.04 (0.69; 1.57)	1.06 (7.00; 1.60)	0.783
	Partner	0.72 (0.38; 1.34)	0.71 (0.38; 1.33)	0.292
	Friends	0.56 (0.18; 1.73)	0.59 (0.19; 1.83)	0.362

**Table 4:** Modified Poisson regression modeling of injecting and other risk factors for HCV infection among PWID in Dar es Salaam, Tanzania

Variable	Category	PR (95%CI)	APR (95%CI)	*p* value
Age at first injection	≤ 15	1	1	
	16–18	0.90 (0.46; 1.78)	0.86 (0.44; 1.69)	0.658
	> 18	0.69 (0.40; 1.21)	0.61 (0.35; 1.07)	0.084
Duration of injection (years)	< 1	1	1	
	1–3	1.92 (0.57; 6.43)	1.99 (0.59; 6.70)	0.265
	4–6	1.76 (0.52; 5.89)	1.81 (0.54; 6.10)	0.338
	7–9	3.34 (1.03; 10.87)	3.41 (1.04; 11.14)	0.041
	10+	3.06 (1.00; 9.41)	2.96 (1.00; 9.13)	0.049
Injection past month, how often	Once/day	1	1	
	> 1 per day	3.20 (0.90; 21.83)	3.25 (1.01; 22.41)	0.037
	Once/week	2.71 (1.00; 20.64)	2.75 (0.93; 21.04)	0.059
	> 1 per week	2.57 (0.84; 18.87)	2.65 (0.80; 19.65)	0.063
Access to clean needle	Yes	1	1	
	No	1.83 (1.54; 11.27)	1.76 (1.44; 12.74)	0.006
Flash blood past month*	No	1	1	
	Yes	2.19 (0.57; 8.44)	2.13 (0.55; 8.13)	0.269
Ever shared needle	Yes	1	1	
	No	0.94 (0.64; 1.36)	0.90 (0.63; 1.38)	0.746
Shared needle past month	No	1	1	
	Yes	1.68 (1.00; 2.94)	1.72 (1.02; 3.00)	0.015
Cleaned last time shared	Yes	1	1	
	No	2.27 (1.01; 6.38)	2.29 (1.00; 6.37)	0.043
On opioid treatment	Yes	1		
	No	0.40 (0.25; 0.64)	0.39 (0.24; 0.63)	< 0.001
Ever sold sex	Yes	1	1	
	No	0.84 (0.55; 1.29)	0.83 (0.55 (1.26)	0.402
Sold sex past month	No	1	1	
	Yes	1.24 (0.80; 1.91)	1.25 (0.81 (1.93)	0.303
Condom use last paid sex	Yes	1	1	
	No	1.77 (0.92; 3.43)	1.87 (1.00; 3.61)	0.050
Ever beaten past year	Yes	1	1	
	No	1.07 (0.73; 1.59)	1.02 (0.69; 1.52)	0.903
Forced sex past year	No	1	1	
	Yes	1.50 (0.81; 2.78)	1.49 (0.80; 2.76)	0.200
Ever arrested past year	No	1	1	
	Yes	1.47 (1.02; 2.12)	1.41 (0.97; 2.05)	0.064
Comprehensive HIV knowledge	No	1	1	
	Yes	1.07 (0.72; 1.57)	1.07 (0.73; 1.56)	0.735
Risk perception	High	1	1	
	Moderate	1.23 (0.72; 2.11)	1.26 (0.73; 2.15)	0.396
	No or low	0.97 (0.65; 1.49)	0.98 (0.65; 1.50)	0.961

Estimation of independent risk factors for HCV infection in a modified multivariable Poisson regression modeling indicated that having injected for 7–9 years (APR, 3.41; 95%CI, 1.04; 11.14) and more than 10 years (APR, 2.96; 95%CI, 1.00; 9.13) were associated with three times higher risk of HCV infection. Similarly, history of injecting several times per day was associated with three-fold the risk of HCV infection (APR, 3.25, 95%CI, 1.01; 22.41).

Lack of access to clean needle when one is in need of one, and sharing needles during the past month, were associated with a 76% (APR, 1.76; 95%CI, 1.44; 12.74) and 72% (APR, 1.72; 95%CI, 1.02; 3.00) increased risk of HCV infection in this population. Additionally, not cleaning a needle the last time it was shared was associated with twice the risk of HCV infection (APR, 2.29; 95%CI, 1.00; 6.37).

Although crude analysis indicated a none statistically significant association, after adjustment of confounders, PWID who did not use a condom the last time they engaged in a transactional sex was associated with 87% increased risk of HCV infection (APR,1.87; 95% CI, 1.00; 3.61). In the contrary, not being enrolled in opioid treatment program was independently associated with 61% decreased risk of HCV infection in this population (APR, 0.39, 955CI: 0.24; 0.63) (Tables 3 and 4).

## Discussion

This study presents the first national estimates of HCV and HIV/HCV co-infection among PWID recruited from the general population in Tanzania. The prevalence’s of HCV antibody and HIC/HCV co-infection were 16% and 43%, respectively, and associated with unsafe injecting behaviors and risk sexual practices.

The prevalence of HCV infection in this study was estimated to be 16.2% and ranged from 13.0 to 20.1%. This estimate is lower than the global prevalence of 52.3% but similar to that of sub-Saharan Africa estimated to be 21.8% [1]. On the other hand, our estimate is more than half (57%) of what was estimated earlier in the city [11]. The observed difference could be explained by differences in the recruitment methods. The previous study included PWID enrolled in methadone substitution therapy who most likely represented a selected population. While duration since injection did not differ between those enrolled and those not enrolled in OST (*p* = 0.104) it is possible that PWID on OST are more likely to have been high-risk injectors who had succumbed adverse consequences forcing them to enroll for treatment. These results, however, differ from what has been published elsewhere [1–3, 20]. Various studies including a Cochrane Review and meta-analysis have provided evidence that OST and NSP are associated with reduced risk of HCV infection [21]. To effectively realize a substantial reduction in HCV infection rates, research and projection models suggest the need to scale up both OST and NSP coverage [13, 22]. In sub-Saharan Africa including Tanzania where OST coverage is still low, these effects may not be evident. Moreover, given that estimates from this study come from the general population recruitment and corroborate estimates from other studies in the region, we believe that these estimates may represent well the magnitude of HCV in this population. It is also worth noting that, having injected during the past 3 months preceding the study was a criterion for recruitment in this study. This means that PWID on OST who have stopped injecting would not be included in this study except for a few who were yet to stop injecting. This explains the reported low proportion of (7%) participants in this study who reported to have been enrolled in OST program in the city.

In July 2018, the World Health Organization released a guideline for care and treatment of persons diagnosed with chronic hepatitis C virus infection [15]. The guideline emphasizes on the need to eliminate hepatitis as a public health threat by 2030. This requires that 90% of people infected with hepatitis to be diagnosed and 80% of those infected to be treated. The guideline identifies PWID as a target population in the current efforts to eliminate HCV infection. While Tanzania has put forward efforts to address the epidemic among PWID through provision of harm reduction intervention such as OST, NSP, and linkage to TB and HIV services, it is high time that these efforts are scaled up and aligned with the WHO recommendation on elimination.

HIV/HCV co-infection was substantially high in this community of PWID compared to what has been published in the USA, Australia, and Canada but lower than those in Caribbean and East Asia and Pacific [3, 23, 24]. While the direct effect of HCV in the natural history of HIV infection remains unclear, HCV has been found to significantly contribute into non-AIDS death among HIV-infected individuals. On the other end, HIV infection affects the natural history of HCV infection through enhanced viral replication, decreased clearance of HCV after acute infection, accelerated liver fibrosis, liver decompensation, and death [4, 10, 25]. The National Guideline for Comprehensive Package of HIV Prevention for Key Population in Tanzania provides guidance on the need to take conscious decision on the selection of ART when treating HIV-infected patients with hepatitis infection including the need for liver function monitoring [26]. Scaling up HIV treatment coverage among PWID will slow down the detrimental effect of HCV infection including the rate of liver fibrosis [25].

Risk factors for HCV infection in this population were mostly related to unsafe injecting behavior and risk sexual behavior. Practice of these behaviors underscores high risk of transmission and acquisition of other blood-borne infection including HIV among PWID. Injecting behaviors identified such as duration one has been injecting, sharing of needles, frequency of injection, and poor access to safe injecting paraphernalia are in line with what has been published earlier nationally and internationally [6, 12, 27–30].

Access to safe and clean syringes and needles is at the cornerstone of disease transmission among PWID. The national prevention package includes provision of safe injecting equipment as a preventive tool but access to such services remains a challenge [28]. It is also important to note that predictors of access to infection prevention commodities are different from predictors of use [29]. Health promotion which impacts knowledge and skills for safer injecting behaviors particularly addressing unsafe sharing of needles should be given priority alongside expanding access.

Selling of sex was common among participants in this study with 69% of PWID indicating to have ever sold sex and half to have done so during the past month preceding the survey. Selling of sex included a large (47%) proportion of male who engaged in transactional anal sex. Of concern was the fact that 70% of those who engaged in transactional sex did not use a condom. Selling of sex among PWID has been reported in many studies and could be related to efforts to secure money to buy drugs [12, 31–33]. Such desperate attempt may obfuscate human judgment for safe sex and lead into unprotected sex. In this study, we found unprotected sex during transactional sex to be associated with 87% increased likelihood of infection with HCV. These findings call for combination of intervention among PWID that addresses injecting and risk sexual behaviors, as well as antiretroviral therapy and Opiate Substitution Therapy [22, 34]. The provision of such combination intervention should be designed not to be an add-on but possibly stepwise to avoid perpetuating risk equivalence beliefs [35].

On one hand, the strength of results emanating from this study lies on the fact that they emanate from a community-based recruitment, include a large sample, included wide selection of seeds, and instituted a careful screening mechanism using experienced former PWID screener and attempts were made to control for biases resulting from the RDS recruitment method. On the other hand, the cross-sectional nature of the design limits the assessment of temporal relationship between estimated risk factor and infection. However, evidence from stronger designs support the causality of the factors identified. Desirability bias emanating from reported sensitive behaviors such as those related to injecting and sexual behaviors may also have affected the findings presented.

## Conclusions

The HIV antibody prevalence among PWID living in the Tanzanian largest metropolitan city of Dar es Salaam was low than global estimates indicating potential for elimination. Risk-injecting behaviors played a major role in HCV infection calling for improved access and use of safe and clean-injecting equipment. Screening for HIV/HCV co-infection should be intensified in HIV Care and Opioid Treatment Programs and other point of care for people injecting drugs. Use of direct-acting antiretroviral treatment would accelerate the achievement of hepatitis infection elimination goal by 2030.

## Data Availability

Data will be available on reasonable request. Please contact the author for data requests.

## Abbreviations

APR: Adjusted prevalence ratio
ART: Antiretroviral therapy
DEFF: Design effect
DFC: Danish foreign cooperation
ELISA: Enzyme-linked immunoassay
HCV: Hepatitis C virus
HIV: Human immunodeficiency syndrome
IQR: Interquarter range
OSP: Opioid substitution therapy
PWID: People who injected drugs
RDS: Respondent-driven sampling

## References

[1] Degenhardt L, Peacock A, Colledge S, Leung J, Grebely J, Vickerman P, Larney S (2017). Global prevalence of injecting drug use and sociodemographic characteristics and prevalence of HIV, HBV, and HCV in people who inject drugs: a multistage systematic review. Lancet Glob Health.

[2] Grebely Jason, Larney Sarah, Peacock Amy, Colledge Samantha, Leung Janni, Hickman Matthew, Vickerman Peter, Blach Sarah, Cunningham Evan B., Dumchev Kostyantyn, Lynskey Michael, Stone Jack, Trickey Adam, Razavi Homie, Mattick Richard P., Farrell Michael, Dore Gregory J., Degenhardt Louisa (2018). Global, regional, and country-level estimates of hepatitis C infection among people who have recently injected drugs. Addiction.

[3] Lavanchy D (2009). The global burden of hepatitis C. Liver Int.

[4] Dore GJ, Freeman AJ, Law M, Kaldor JM (2002). Is severe liver disease a common outcome for people with chronic hepatitis C?. J Gastroenterol Hepatol.

[5] Te HS (2010). JD: epidemiology of hepatitis B and C viruses: a global overview. Clinical Liver Disease.

[6] Mmbaga EJMK, Makyao N, Leshabari M (2017). Prevalence and predictors of human immunodeficiency virus and selected sexually transmitted infections among people who inject drugs in Dar es Salaam, Tanzania: a new focus to get to zero. Sex Transm Dis.

[7] Chen JY, Feeney ER, Chung RT (2014). HCV and HIV co-infection: mechanisms and management. Nat Rev Gastroenterol Hepatol.

[8] Mandorfer MSP, Steiner S, Reiberger T, Peck-Radosavljevic M (2016). Advances in the management of HIV/HCV coinfection. Hepatol Int.

[9] BM BY, Di Martino V, Charlotte F (1999). Azria F Liver fibrosis progression in human immunodeficiency virus and hepatitis C virus coinfected patients. Hepatology.

[10] Pineda JA, Romero-Gómez M, Díaz-García F, Girón-González JA, Montero JL, Torre-Cisneros J, Andrade RJ (2005). HIV coinfection shortens the survival of patients with hepatitis C virus-related decompensated cirrhosis. Hepatology.

[11] Lambdin BH, Lorvick J, Mbwambo JK, Rwegasha J, Hassan S, Lum P, Kral AH (2017). Prevalence and predictors of HCV among a cohort of opioid treatment patients in Dar es Salaam, Tanzania. Int J Drug Policy.

[12] Mohamed Zameer, Rwegasha John, Kim Jin U., Shimakawa Yusuke, Poiteau Lila, Chevaliez Stéphane, Bhagani Sanjay, Taylor-Robinson Simon D., Thursz Mark R., Mbwambo Jessie, Lemoine Maud (2018). The hepatitis C cascade of care in people who inject drugs in Dar es Salaam, Tanzania. Journal of Viral Hepatitis.

[13] Natasha K Martin KJ, Matthias an der Heiden, Christoph Boesecke, Anders Boyd, Knud Schewe, Axel Baumgarten, Thomas Lutz, Stefan Christensen, Alexander Thielen, Stefan Mauss, Jürgen K Rockstroh, Britt Skaathun, Patrick Ingiliz: Eliminating hepatitis C virus among human immunodeficiency virus–infected men who have sex with men in Berlin: a modeling analysis. J Infect Dis 2019, 220(10):1635–1644. 10.1093/infdis/jiz367 PMC7360352 31301142

[14] Nguyen JBA, Jhaveri R (2019). Cost effectiveness of early treatment with direct-acting antiviral therapy in adolescent patients with hepatitis C virus infection. J Pediatr.

[15] Organization. WH (2018). Guidelines for the care and treatment of persons diagnosed with chronic hepatitis C Virus Infection.

[16] Chaillon AMS, Hoenigl M, Solomon SS, Vickerman P, Hickman M, Skaathun B, Martin NK. Cost-effectiveness and budgetary impact of HCV treatment with direct-acting antivirals in India including the risk of reinfection. PLoS One. 2019;14(6):e0217964. 10.1371/journal.pone.0217964.eCollection.2019. 10.1371/journal.pone.0217964 PMC6553784 31170246

[17] Statistics NBo: Tanzania HIV Impact Survey 2016-2017: Preliminary findings. In. Dar es Salaam: NBS, NACP; 2017.

[18] Wejnert C (2009). An emprerical test of respondent-driven sampling: point estimates, variance, degree measures, and out-of equilibrium data. Sociol Methodol.

[19] Heckathorn D (1997). Respondent-driven sampling: a new approach to the study of hidden populations. Soc Probl.

[20] Bernd Schulte CSS, Strada L, Rosenkranz M, Schäfer I, Verthein U, Reimer J. Hepatitis C virus prevalence and incidence in a large nationwide sample of patients in opioid substitution treatment in Germany: a prospective cohort study. Clin Infect Dis. 2019; 10.1093/cid/ciz661. 10.1093/cid/ciz661 31631215

[21] Platt LMS, Reed J, Vickerman P, Hagan H, French C, Jordan A, Degenhardt L, Hope V, Hutchinson S, Maher L, Palmateer N, Taylor A, Bruneau J, Hickman M (2018). Needle and syringe programmes and opioid substitution therapy for preventing HCV transmission among people who inject drugs: findings from a Cochrane Review and meta-analysis. Addiction.

[22] Martin NK, Hickman, M., Hutchinson, S. J., Goldberg, D. J., & Vickerman, P: Combination interventions to prevent HCV transmission among people who inject drugs: modeling the impact of antiviral treatment, needle and syringe programs, and opiate substitution therapy. Clin Infect Dis 2013, 57(suppl 2):S39–S45. doi:10.1093/cid/cit1296. 10.1093/cid/cit296 PMC3722076 23884064

[23] Maher L, Jalaludin B, Chant GK, Jayasuriya R, Sladden T, Kaldor MJ, Sargent PL (2006). Incidence and risk factors for hepatitis C seroconversion in injecting drug users in Australia. Addiction.

[24] GJ MHK, Flaxman AD, Wiersma ST (2013). Global epidemiology of hepatitis C virus infection: new estimates of age-specific antibody to HCV seroprevalence. Hepatology.

[25] Bräu NSM, Ríos-Bedoya CF, Fernández-Carbia A, Paronetto F, Rodríguez-Orengo JF, Rodríguez-Torres M (2006). Slower fibrosis progression in HIV/HCV-coinfected patients with successful HIV suppression using antiretroviral therapy. J Hepatol.

[26] Programme NAC: Comprehensive Guideline for HIV prevention among Key Population in Tanzania. . In*.* Dar es Salaam, NACP; 2016.

[27] Dahoma MJSA, Abdool R (2006). HIV and substance abuse: the dual epidemics challenging Zanzibar. Afr J Drugs Alcohol Studies.

[28] Lambdin Barrot H., Bruce R. Douglas, Chang Olivia, Nyandindi Cassian, Sabuni Norman, Zamudio-Haas Sophia, McCurdy Sheryl, Masao Frank, Ivo Yovin, Msami Amani, Ubuguy Omar, Mbwambo Jessie (2013). Identifying Programmatic Gaps: Inequities in Harm Reduction Service Utilization among Male and Female Drug Users in Dar es Salaam, Tanzania. PLoS ONE.

[29] Mlunde LBSB, Mbwambo JK. Correlates of health care seeking behaviour among people who inject drugs in Dar es Salaam, Tanzania. Int J Drug Policy. 2016;30. 10.1016/j.drugpo.2015.12.012:66-73. 10.1016/j.drugpo.2015.12.012 26821555

[30] Ross MWMS (2008). Kilonzo GP drug use careers and blood-borne pathogen risk behavior in male and female Tanzanian heroin injectors. Am J Trop Med Hyg.

[31] MS REA, Mbwambo JK, Lambdin BH, Voets A, Pont S, Maruyama H, Kilonzo GP. An overview of HIV prevention interventions for people who inject drugs in Tanzania. Adv Prev Med. 2013;183187. 10.1155/2013/183187):183-187. 10.1155/2013/183187 PMC3549374 23346410

[32] Strathdee SASJ (2010). Epidemiology of HIV among injecting and non-injecting drug users: current trends and implications for intervention. Current HIV/AIDS Report.

[33] Zamudio-Haas Sophia, Mahenge Bathsheba, Saleem Haneefa, Mbwambo Jessie, Lambdin Barrot H. (2016). Generating trust: Programmatic strategies to reach women who inject drugs with harm reduction services in Dar es Salaam, Tanzania. International Journal of Drug Policy.

[34] Doyle Joseph S., Aspinall Esther J., Hutchinson Sharon J., Quinn Brendan, Gore Charles, Wiktor Stefan Z., Hellard Margaret E. (2015). Global policy and access to new hepatitis C therapies for people who inject drugs. International Journal of Drug Policy.

[35] Harris MRT (2013). Injecting practices in sexual partnerships: hepatitis C transmission potentials in a ‘risk equivalence’ framework. Drug Alcohol Depend.

